# Targeting tumor innervation: premises, promises, and challenges

**DOI:** 10.1038/s41420-022-00930-9

**Published:** 2022-03-25

**Authors:** Xinyu Li, Xueqiang Peng, Shuo Yang, Shibo Wei, Qing Fan, Jingang Liu, Liang Yang, Hangyu Li

**Affiliations:** Department of General Surgery, The Fourth Affiliated Hospital, China Medical University, Shenyang, 110032 China

**Keywords:** Cancer, Cell biology

## Abstract

A high intratumoral nerve density is correlated with poor survival, high metastasis, and high recurrence across multiple solid tumor types. Recent research has revealed that cancer cells release diverse neurotrophic factors and exosomes to promote tumor innervation, in addition, infiltrating nerves can also mediate multiple tumor biological processes via exosomes and neurotransmitters. In this review, through seminal studies establishing tumor innervation, we discuss the communication between peripheral nerves and tumor cells in the tumor microenvironment (TME), and revealed the nerve-tumor regulation mechanisms on oncogenic process, angiogenesis, lymphangiogenesis, and immunity. Finally, we discussed the promising directions of ‘old drugs newly used’ to target TME communication and clarified a new line to prevent tumor malignant capacity.

## Facts

- Innervation in some solid tumor is associated with poor outcome.
- As a malignant factor, intratumoral nerve play an important role in tumor progression, and its density determines tumor malignant capacities.
- Nerves in tumors can regulate various biological process, such as angiogenesis lymphangiogenesis, immunity and inflammation, Fibroblasts and the extracellular matrix, DNA repair, and oncogene activation.
- Otherwise, tumor cells also involved in axonogenesis, neurogenesis, and neural reprogramming process in tumor microenvironment.
- Targeting innervation provides a huge research prospect for tumor therapy.

## Open questions

- How to achieve clinical treatment through cutting off nerves in solid tumors?
- How to target nerve-tumor communication to prevent tumor prorgression?
- What is the major biomolecule in tumor microenvironment mediating axonogenesis, neurogenesis, and neural reprogramming process?
- Whether clinical neurologic drugs can be a way to treat tumors?

## Nerves step into our view

Cancer is a major health problem with an increasing impact on all societies. In 2018, there were an estimated 18.1 million new cancer cases and 9.6 million cancer-related deaths globally [1]. Solid cancers develop through primary cancer cells that accumulate genetic mutations as they grow, often acquiring aggressive/metastatic characteristics. However, metastases can occur without the formation of a primary tumor. In fact, the control mechanisms of primary and secondary cancers (proliferation and invasion/metastasis, respectively) are different, and at least partially independent [2, 3]. Pathophysiology is extremely complex and variablein space and time and includes various factors when considering treatment. A large part of this complexity is due to the epigenetic nature of cancers, which gives cancer the ability to withstand constant changes [4]. In addition, cancer cells have a considerable amount of “stemness”; in fact, many embryonic genes, including splice variants for development regulation, are expressed in cancer cells [5, 6]. Unfortunately, owing to the emergence of drug resistance, the most commonly used cancer treatments have significant limitations, including side effects such as toxicity and damage to peripheral tissues, as well as high treatment costs. From primary diagnosis to advanced treatments in clinical management, the present needs have not been met. Therefore, efforts are being made to identify functional markers and cancer development mechanisms that will contribute to early diagnosis and effective long-term treatment.

A growing body of evidence has suggested that cancer prognosis is related to intratumoral nerve infiltration. This phenomenon occurs in organs or tissues with high innervation, especially in pancreatic cancers(PRC) (nearly 100%), 80% of head and neck cancers, 75% of prostate cancers (PC), and 33% of colorectal cancers (CRC) [7]. The density of infiltrated nerves is positively associated with tumor metastasis, morbidity, and mortality, and has been shown to be an independent risk factor for survival prognosis in a variety of cancers, including PC [7–10], gastric cancer(GC) [11, 12], biliary tract cancer [13], head and neck cancer [14–16], and cervical cancer [17, 18]. In PRC [19, 20], PC [21], GC [22], and CRC [23–26], the density of infiltrated nerves is a factor that relates to recurrence risk. Furthermore, innervation of cancers may play an important direct role in promoting metastasis, as cancer-associated nerves may extend into the central nervous system and activate the precursors of metastasis [23, 27].

Driven by evidence from clinical studies, new translational cancer therapy techniques are being developed to target the interaction between nerves and the TME. In this review, we summarized the role of cancer cells in regulating neurogenesis and described how nerves modulate the TME to promote cancer development and progression. Our overarching aim was to thereby help clinicians and researchers gain a deeper understanding of the mechanisms of nerve-cancer interactions.

## Brief description of nerves in cancer progression

Since the 16th century, nerves and blood vessels have been observed to move at the same time, and both structures are necessary for maintaining organs [28]. Because cancers need these blood vessels and nerves to grow and survive, it was inferred that crosscutting of nerves could be used to control cancer progression. This hypothesis was first tested by surgeons who attempted to treat lip cancers by crosscutting the trigeminal nerves and its accessory blood vessels [28]. Although the transection of nerves and blood vessels achieved symptom control (reducing ulcers and pain), all four patients in the study eventually required surgical removal of their pathological tissue. Despite this advancement, the molecular mechanisms and roles of nerves in the TME remain to be an unelucidated question until the last 10 years. Although the role of denervation in reducing tumor growth has been reported, the progression of cancer-nerve interaction is still slow [29–32]. Unlike cancer perineural invasion, this phenomenon of cancer-nerve tendency as observed in earlier studies is cancer cells disseminate through lymphatic vessels within the perineural space, it is a direct reciprocal process [33]. Intriguingly, several studies had found that perineural space lacks lymphatic vessels [34, 35]. This means that cancer cells in this circumstance spread in the perineural space, itmay be a purely mechanical concept, because along perineural sheath, cells can have the least amount of resistance [36, 37].

## Cancer-nerve regulation

**Fig. 1:**
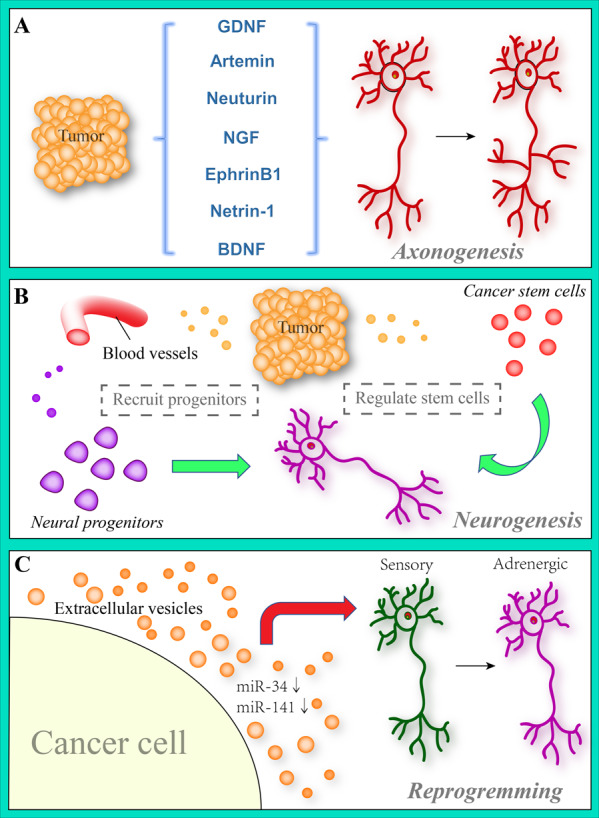
Tumor-mediated nerve neurogenesis, axonogenesis and reprogramming in the TME. **A** During tumor progression, neurogenic factors, such as GDNF, BDNF, NGF, Artemin, Neuturin, EphrinB1, and Netrin-1, are released by tumor cells, inducing the axonal growth of nerves. Neurotrophin binding to its cognate receptors on nerves affects axonogenesis. **B** Tumor cells can recruit neural progenitors into the tumor stoma via the circulatory system, where they are stimulated by TME factors to differentiate into mature nerves. In addition, nerves also can directly regulate cancer stem cells via multiple signals. **C** Prior research has suggested that cancer-related extracellular vesicles play an important role in this reprogramming process.

As tumor growth requires increased angiogenesis, increased vascular density is one of the earliest histological changes observed in tumor tissues. Previous study showed that as cancer progresses from preneoplastic lesions to dominant cancers, nerve density can almost double to the level seen in the non-neoplastic control group [38, 39]. Just as wires supply electricity to electrical circuits, nerves can be thought of as providing information inputs to tissues. The denervated mode stops this signaling mechanism and is thus considered to be an effective way to treat tumors (Fig. 1).

### Cancer-related axonogenesis

Co-culture experiments revealed that the dorsal root ganglia of mice form bumps on prostate cancer cells, and promote the differentiation of nerve cells to cancer cells (cancer cell migration to the nerve was also observed), which reduces the caner-nerve distance, thus promoting the formation of a cancer-nerve crosslinking system [36]. Formation of axons in the organs and tissues increase the nerve density. Through two-dimensional and three-dimensional reconstruction of the prostate, Ayala et al. found that the nerve density and size in the tissues of prostate cancer patients increased significantly, and confirmed the formation of cancer-related nerve axons. Axonogenesis has been observed in preneoplastic lesions of the prostate and may be an early event or a key factor supporting prostatic cancer initiation [38]. In addition, the same study also found that the axon guidance molecule semaphorins, which is the largest and most important family in axonogenesis, in the study, semaphoring 4 F was found highly expressed in DU-145 cells and could be secreted by these cells when co-cultured with PC-12 cells. It releases potential abilities may via binding with PDZ (for PSD95, Discs-large, and ZO1)-domain binding sequence of neuropilins (NPs) [40]. Compared to the negative control group, S4F induced neurite sprouting and increased neurite length by nearly three-fold. Another study observed that small interfering RNA (siRNA) inhibitors of S4F reduced neuronal overgrowth and neurite sprouting [38]. This was the first time that cancer cells were shown to secrete a known neurotropic molecule that promotes axonogenesis. Intriguingly, CD72 and Tim2, which are the immune-related molecules that were found to bind with semaphorins, although it has a low affinity, it still promotes the activation and differentiation of T cells [41, 42]. This noted that semaphorins possess multiple functions not only in the axonogenesis but involve in immune system.

Subsequent breast cancer studies also demonstrated that breast cancer cells promoted axonogenesis. In the N-methyl-N-nitrosourea (MNU)-induced breast cancer rat model, immunofluorescence experiments showed that axons were generated in breast cancer tissues with increased nerve density. Interestingly, sensory and sympathetic nerves were detected in cancer tissues, but parasympathetic nerves were not observed [43, 44]. The distribution of sensory nerves and dysregulation of the sympathetic-parasympathetic nerve are closely related to the occurrence and progression of cancers. Head and neck squamous cell carcinoma [45], pancreatic duct adenocarcinoma [46], cervical carcinoma [47], and basal cell carcinoma [48] all show dependence on sensory nerves. As mentioned earlier, prostate cancer cells induce sympathetic axonogenesis, and as a reciprocal process, sympathetic activation induces adrenergic receptor activation, which in turn accelerates tumor growth, while intratumoral sympathetic activation enhances cell resistance to cytotoxic chemotherapeutic agents [49–51]. According to the above studies, cancer-associated axonogenesis is mostly observed for the sensory and sympathetic nerves, and the impact of cancer cells on parasympathetic axonogenesis requires further study and exploration. These studies also suggested that sympathetic signaling may be a potential therapeutic target, and cancer-related axonogenesis may also drive cancer progression. However, the question of how to transfer information through the interaction between them remains unanswered. An in-depth study of exosomes may provide good results in this regard.

In a study on the regulation of axonogenesis by tumor cells, Paola et al. found that cancer cells with impaired exosome release had less effect on nerves compared with the control group in vivo, and that inhibition of exosome release also attenuated cancer innervation [52]. EphrinB1 in exosomes significantly promoted axonogenesis and cancer innervation in vitro and in vivo, whereas loss of ephrinB1 did not cause this phenomenon [52]. Exosomes in HPV-positive oral squamous cell carcinoma with higher ephrinB1 expression showed a greater capacity for axonogenesis, than HPV-negative carcinoma exosomes. Similar results were observed in colorectal cancer cell lines, melanoma cell lines, and breast cancer cell lines (all three exosomes were labeled with CD9) [52]. EphrinB1 can active Eph receptor tyrosine kinases, and induce the activation of MAPK signaling [53], but in axonogenesis process, the sense of ephrinB1-related MAPK activation is a puzzle.

Taken together, these results suggest that CD9^+^ exosomes released by HPV-positive cancer cells have a greater ability to promote cancer innervation in vivo [52]. Exosomes from other cancer cell lines also showed evidence of promoting axonogenesis in vitro, suggesting that in other solid cancers, cancer innervation may be induced by cancer-released exosomes, thus providing abundant blood supply through axonogenesis and promoting the growth of cancers [52]. Consistent with this, ephrinB1 has been found to exhibit angiogenic properties in several previous studies [54, 55]. Studies have also found that nerves and blood vessels exhibit a certain degree of distribution similarity [43]. In addition, a recent study found that hypoxia induced erythropoietin (EPO)/EPOR-related dendritic spine density, which is may function via pre-transcriptional regulation because of the EPO and EPOR transcripts in pyramidal neurons of the hippocampal CA1 region [56]. It was mentioned that one of the hallmarks of tumors is that it has lots of hypoxia regions in its microenvironment [57, 58], therefore, hypoxia in TME to regulate axonogenesis is still unclear, in combination with the foregoing, in some innervated tumors, such as PC, CRC and so on, hypoxia may make great effect in axonogenesis.

All these studies suggested a certain correlation between nerves and axonogenesis; however, regardless of the combination of relevant factors, interference with cancer exosome release or blockage of the response ability of nerves to exosomes may prove to be an effective cancer treatment strategy. Although this concept needs to be rigorously tested, if proven to be effective, it has the potential to drive an important clinical transformation.

### Cancer drives neurogenesis

In addition to research on axonogenesis, Ayala et al. proposed that cancer cells could promote neurogenesis [38]. In prostate cancer, an increase in the number of neurons in the ganglion indicates the occurrence of a neurogenetic process underlying cancer development and progression [38]. However, it is worth noting that this process may be more cancer-specific. In healthy prostates, sympathetic nerves can regenerate after injury; studies have speculated that this may be a result of axonogenesis, with no evidence of neurogenesis [59]. In prostate cancer, compared to normal prostate tissues, the penetration of newly formed autonomic nerves into the tumors contributes to the initiation and progression of cancers through activation of beta-adrenergic and toxicological cholinergic signaling, respectively [60, 61]. DCX is a typical cellular marker of neural precursors located in the developing central nervous system and adult neurogenic regions [62–65]. The stroma of human prostatic primary cancers contain DCX-positive cells, which also express specific markers of neural progenitor cells, such as PSA-NCAM [66] and Internexin [67, 68]. Mauffrey et al. showed that neural progenitor cells migrate through the bloodstream from the neurogenic region in the subventricular region to cancerous and metastatic sites, where they differentiate into a mature adrenergic neuro-phenotype [69]. Prostate cancer patients were divided into high-and low-risk groups, in which the density of DCX-positive cells was strongly associated with cancer invasion and recurrence, and DCX-positive progenitor cells initiated neurogenesis and could differentiate into adrenergic nerves, involved in the malignant progression of cancers, but the specific mechanisms about how to recruit cells and achieve differentiation is still a puzzle [37, 69]. This research shed light on the interaction between the central nervous system and prostate cancers and reveals the unique migration and orientation of central nervous precursor cells that can differentiate into adrenergic neurons, which promote the development and metastasis of the primary tumor. Similarly, in lung adenocarcinoma, this process can also increase the bone matrix activity without provoking bone metastasis, and drives the bone neutrophil response from a distance [70].

These studies illustrated how cancers communicate with distant organs and tissues to recruit the cells that they need, and highlight the importance of neurogenesis. However, it is worth noting that cancers can deplete normal cells in the same manner; indeed, several clinical oncology studies have clearly shown that patients who receive chemotherapy are at a greater risk of experiencing brain nerve progenitor cell damage and cognitive decline; however, in patients who do not receive chemotherapy, the depletion of neural progenitor cells by cancer cells may also lead to cognitive impairment [69]. These results open a novel avenue for diagnosing and monitoring the development of cancers and suggest the therapeutic potential of inhibiting the migration of neural progenitor cells to cancer cells and targeting neural progenitor cells in the TME.

In addition to remote regulation, cancer can also form new neurons from cancer stem cells. Lu et al. [71] obtained cancer stem cells (CSCs) from patients with gastric cancer and colorectal cancer tissues, and through subcutaneous injection and intraperitoneal injection constructed the models, it were measured by immunofluorescence, and the results showed the cells to be positive for the nerve marker MAP in vivo, indicating that a subset of the monoclonal CSC cells can be induced to differentiate into nerve cells in vitro, partly differentiated sympathetic neuronal marker tyrosine hydroxylase (TH) in colorectal and gastric CSCs, differentiated gastric CSCs had cells that expressing the parasympathetic marker vesicular acetylcholine transporter (VaChT) [71]. These data suggest that human gastric and colorectal CSCs can generate functional autonomic nerve cells, and that the emergence of such new nerve cells is partly dependent on the differentiation of CSCs. Weakening the ability of CSCs to produce neurons can inhibit the growth of cancer to some extent. In addition, the ability of CSCs to transdifferentiate into the vascular endothelium may be one of the mechanisms underlying cancer angiogenesis and drug resistance [72]. Therefore, it is necessary to target the differentiation of CSCs into neurons and to interfere with the differentiation of CSCs in other directions in order to prevent cancer resistance to anti-neuro/vascular therapy.

### Cancer induces neural reprogramming

Studies have found that exosome-induced neural reprogramming is usually observed during cancer development, suggesting that cancer-derived extracellular vesicle (EV) function in cancer-associated axonogenesis. Exosomes of p53-depleted head and neck cancer cells can carry multiple miRNAs to participate in axonogenesis, while p53 knockout or mutation (p53^C176F^ and p53^A161s^) increases the number of nerve fibers [45]. RNA sequencing analysis of exosome miRNAs revealed that the differentially expressed genes were highly associated with neuronal growth, morphogenesis, synaptogenesis, differentiation, stemness, and synaptic transmission [45]. It obviously increased the density of tyrosine hydroxylase (TH, adrenergic nerves) positive fibers in TP53-Mut or deficient OCSCCs compared to TP53-WT, while the density of vesicular acetylcholine transporter (parasympathetic nerves) positive fibers was almost unchanged [45]. The aggregation of adrenergic neurons is associated with extracellular vesicles originating from p53-deficient cancer cells. Furthermore, several in vivo studies have shown that norepinephrine secretion can be detected after intratumoral injection of p53-deficient EVs, suggesting that p53-deficient exosomes can induce neural reprogramming, while knockout of Rab27A and Rab27B inhibits the reprogramming process [45]. Rab27A and Rab27B are key proteins in the EV release process [73, 74]; EVs can participate in cell-to-cell communication and in the regulation of TME; thus, interfering with these Rab proteins would compromise the vesicle transport and selective loading process, and may provide an effective way to prevent malignant manifestation of cancers [75]. Sequencing and analysis of miRNAs in EVs revealed that the expression of miR-34a and miR-141-5p in EVs of OCSCC with p53 deletion was significantly reduced, and knockdown of miR-34a and miR-141-5p in wild-type p53 cell EVs could lead to similar results as those in the p53 deletion group. However, miR-141-5p had only a weak effect, suggesting that miR-34a and miR-141-5p in EVs are involved in the neural reprogramming process, while miR-34a plays a major role [45]. Furthermore, reduced miR-34a levels not only promoted neurogenesis of sensory nerves, but also induced the trans-differentiation of nerves that can produce norepinephrine [45].

Previous studies have shown that miR-34a plays an important role in the differentiation of mesenchymal stem cells (MSCs) and CSCs [76–78]. Intriguingly, miR-34a is highly expressed in the adult mammalian brain and has been shown to be involved in a range of neurodevelopmental and neuropathological processes. MiR-34a regulates neural stem cell/progenitor cell differentiation and neurogenesis [79]. These results suggest a novel potential mechanism by which tumor cells reprogram the nerve cells in the microenvironment.

Here, as an extension, we noted that knockdown of miR-34a not only induced neural reprogramming, but also promoted sensory neurogenesis. Based on a physiological nerve distribution feature, the oral trigeminal nerve is widely distributed, and oral cancer-related pain is the most frequent symptom [45, 80–82]. Therefore, miR-34a may have some significance in alleviating cancer-related pain in oral cancer patients based on the inhibition of nerve infiltration; however, the difference in physiological characteristics and other features between different types of cancers should be noted. For example, the autonomic nerve plays a dominant role in TME of prostate cancer, pancreatic cancer, breast cancer, among others, but its role is less well established in other cancers [28].

## The role of the nerve in tumor biology

Thus far, we have summarized the regulation of cancer cells by neighboring nerve cells, but the influence of nerves on cancers is extensive and complex, and the initial research on the neurological impact on cancer biology was inspired by clinical observations indicating a potential link between stress and cancer progression [83–85]. People subjected to repeated stress are susceptible to disease due to abnormalities in the peripheral stress response system, including the hypothalamic-pituitary-adrenal axis and glucocorticoids, as well as the sympathetic-adrenal axis [86]. The sympathetic nervous system (SNS) regulates the function of almost all human organs through the release of catecholamines from the local SNS or adrenal system into the circulatory system [86–89]. Recent pharmacoepidemiological data have shown a reduction in adverse events in cancer patients administered beta-adrenergic antagonists (also known as beta-blockers) prior to diagnosis [90–93].

**Fig. 2:**
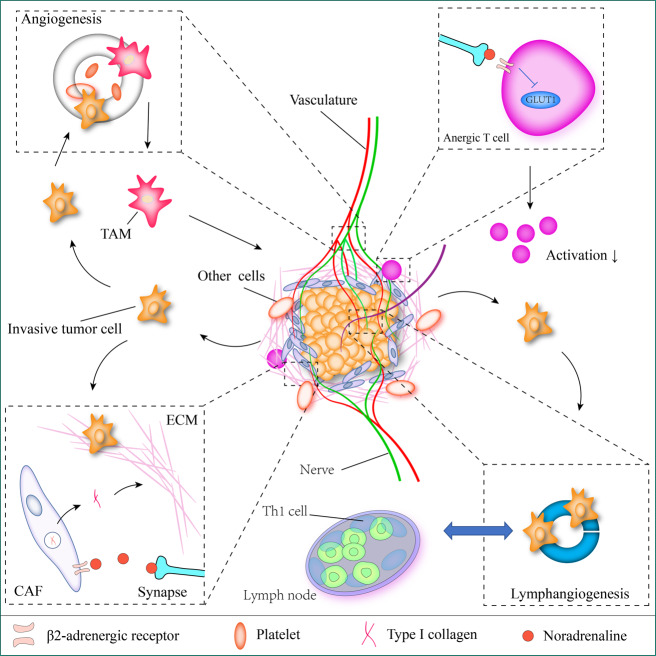
Nerve cells drive tumor progression and extracellular matrix (ECM) remodeling. Nerves interact with multiple stromal and malignant components that promote tumor growth and dissemination during tumor development. Signaling from adrenergic nerves stimulates angiogenesis and lymphangiogenesis and activates tumor metastatic pathways. This signaling also activates T cells by inhibiting glucose transporter (GLUT1) expression, which in turn results in a decrease in metabolic activation. In addition, adrenergic nerves help in maintaining the immunosuppressive microenvironment by recruiting T helper 1 cells (Th1 cells) to tumors. Noradrenaline (NA) induce cancer-associated fibroblasts (CAFs) to produce type I collagen that remodel ECM.

Experimental analyses of animal models in vivo have shown that behavioral stress can accelerate the progression of various cancers, including breast cancer, prostate cancer, ovarian cancer [94–97], neuroblastoma [98, 99], melanoma [100, 101], pancreatic cancer [102] and some hematopoietic system cancers (such as leukemia) [103, 104]. The mechanisms of cancer progression are also involved DNA repair, oncogene activation, angiogenesis, lymphangiogenesis, immunity and inflammation, fibroblasts, the extracellular matrix, among others (Fig. 2). Both the central nervous system and the nervous cells in the TME can exert significant impacts on cancers [105]. In this section, we discuss the impact of nerves on the individual components of the TME.

### Angiogenesis

Angiogenesis, the formation of new blood vessels from the existing vascular system, is necessary for cancer growth [106]. In tissue matrix composition, adrenergic nerves closely related to blood vessels (mainly arterioles and capillaries) and nerve fiber orderly distribution (side by side) in tissues, the connection mechanism of nerve and vascular network seems to have some deep similarities, and axon guidance molecules are involved in this common regulation model [107, 108]. A recent study showed that adrenergic neuro-derived norepinephrine activates the endothelial β-adrenergic receptor signaling pathway in prostate cancer and is a key factor in promoting the exponential proliferation of cancer cells [39]. Deletion of ADRB2 (the gene encoding the β2-adrenergic receptor) inhibits angiogenesis by enhancing oxidative phosphorylation in endothelial cells. Codeletion of Adrb2 and Cox10, which encodes the cytochrome IV oxidase assembly factor, can prevent the metabolic transformation caused by Adrb2 deletion, and thus rescue the progression of prostate cancer [39]. Neurochemical factors, such as catecholamines help to active ADRB2, and activation of the endothelial cells cyclic AMP (cAMP)-protein kinase A (PKA) signaling pathway [97], which is a crucial promoter in angiogenesis via multiple pathways, such as Notch and autophagy [109, 110]. This provides a novel strategy that by targeting cAMP signal will make a prospective way to prevent angiogenesis in TME. During tissue development and cancer progression, endothelial cells often rely on glycolytic metabolic procedures for directed cell migration, which is necessary for angiogenesis [111, 112]. The fluorescent co-localization of nerves (TH^+^) and endothelial cells (CD31^+^) was significantly increased in tissues with high-grade prostate intraepithelial neoplasia, suggesting that a shortened physical distance between nerve cells and endothelial cells is associated with an increased malignant capacity of prostate cancer. Interestingly, within the first 18 days of neoplasia, cancer tissues in the control group and the Adrb2 receptor knockout group showed no difference in size, vascular permeability, or hypoxia, whereas after 18 days, the difference was significant [39].

### Lymphangiogenesis

The lymphatic system plays an important role in immune function and can thus influence the course of the disease. Physiologically, the lymphatic system maintains internal environmental stability by guiding cells and solutes from the surrounding interstitial fluid to the lymph nodes via the lymphatic vessels, where they perform immune examinations [113, 114]. In addition, the lymphatic system helps eliminate inflammation by transporting immune cells away from the site of infection [115]. In cancers, the lymphatic system promotes disease progression by providing pathways for the escape of cancer cells, and is also a rich source of chemokines that promote the aggressive properties of cancer cells [114, 116, 117]. The lymphatic system is similar to the vascular system, which is highly innervated by adrenergic nerves and SNS fibers [118]. Acute SNS activity has been shown to increase lymphatic contraction [119, 120] and lymphocyte output to the lymphatic circulation [121]. In situ and transgenic models of breast cancer, stress promotes the release of norepinephrine in the nervous system and activates the release of cancer-derived VEGFC to promote lymphangiogenesis and remodeling, and it also increased the expression of VEGFR3(FLT4) in stoma cells [122]. In addition, β-adrenergic receptor signaling promotes the macrophage expression of COX2 and increases the secretion of PGE2, which thereby activates the release of cancer-derived VEGFC to facilitate metastasis [122]. It notes that in lymphangiogenesis process, nerves-derived catecholamines may play positive role through inflammation signal and VEGFC/FLT4. Similar to its effect on angiogenesis, sympathetic denervation has been shown to reduce lymphatic vessel formation, which is associated with reduced cancer aggression [123]. The deep exploration found beta-blockers reduced the risk of lymph node and distant metastasis [122], which provide a clinical treatment direction to decrease the lymph node and distant metastasis.

### Immunity and inflammation

Lymphocyte activation and infiltration of cancers are key components of the host’s anticancer immune response [124]. Stress increases the activation of lymphocytes and produces pro-inflammatory cytokines such as interleukin-6 (IL-6) [125]. The expression levels of norepinephrine and IL-6 in ovarian cancer tissues of stressed patients were significantly increased compared to those in non-stressed patients (matched for age and disease stage) [126]. In vitro studies have shown that norepinephrine promotes the release of the pro-inflammatory cytokines IL-6 and IL-8 from ovarian cancer cells by acting on the β2-adrenergic receptor signaling pathway [126, 127]. However, highly innervated tissues exhibit low levels of activated T helper cells [124, 128, 129]. Activation of β2-adrenergic receptors can inhibit GLUT1 expression to reduce glucose uptake by CD8^+^ T cells, thereby inhibiting its activation, leading to immune escape via immunosuppression, in addition, adrenergic signaling activation impairs CD8^+^ T-cell mitochondrial function and mass [130]. It means that adrenergic signaling tightly links to the T cell metabolism, and related to immune activation. Besides that, increasing of TNF in the TME contributes to cancer-associated macrophage recruitment, and stress induced adrenergic nerve activation led to a significant increase in intratumoral TAM in a xenograft model of breast cancer [96]. Interestingly, regardless of TAMs, tumor-infiltrating lymphocytes (TILs), or myeloid-derived suppressor cells (MDSCs) expressed β2-adrenergic receptors, cancer growth was slowed, and plasma immunosuppressive molecules were reduced after knockout of the MDSC ADRB2 gene in breast cancer transgenic mice [131]. Therefore, this still needs to be further explored the mechanisms of nervous signalings in immune system in TME. In a word, denervation or cancelation of adrenergic signaling may provide novel approaches to improve the immunotherapeutic response in highly innervated cancers.

### Fibroblasts and the extracellular matrix

Changes in the three-dimensional structure and composition of the TME greatly affect cancer progression and metastasis [132–134]. For example, in many cancers, dense extracellular matrix (ECM) deposition is a part of the promotive connective tissue response, which acts as a physical and chemical barrier against immune cell infiltration, creating an immune-privileged environment [133]. Simultaneously, the composition of the ECM changes to a type I collagen-rich environment, forming an angiogenic super-polymer that assists in the migration of new blood vessels and nerves [135–138]. Furthermore, although an increase in ECM density helps to block immune cell entry in the early stages of cancers, ECM degradation by matrix metalloproteinases (MMPs) during the late metastatic phase of disease progression allows cancer cells to migrate and spread [139]. Collagen remodeling is key to the spread of cancer in the later stages of the disease. In an in situ mouse model of PDAC, stress induced adrenergic signaling increased MMP expression in the stromal compartment more than 100 fold and further increased metastasis, while inhibition of the β-adrenergic receptor with propranolol suppressed this phenotype [102]. Similarly, in an in situ mouse model of breast cancer, adrenergic signaling in the stroma increased collagen remodeling through adrenergic receptors, thereby enhancing metastasis, while a decrease in norepinephrine inhibited this process [140].

### DNA repair

The β-adrenergic signaling pathway can inhibit DNA damage repair and p53-related apoptotic processes [99, 141–143], suggesting that increased SNS activity may promote tumor initiation or chromosomal instability. Long-term activation of adrenergic signaling can lead to ARRB1/AKT-mediated MDM2 activation [141]. As an E3 ligase, MDM2 promotes the rapid degradation of p53 via interaction with p53, and suppresses its transcription [144]. Adrenergic signaling activates MDM2 to promote the rapid degradation of p53 in the thymus and testis, resulting in the accumulation of damaged DNA, and increasing the probability of chromosomal aberration in tissues; administration of the β-receptor antagonist propranolol blocked this process [141]. Similar results were found in breast cancer, where propranolol effectively increased the expression of p53 and inhibited the growth of cancer cells [145]. However, it is worth noting that neural distribution differs significantly according to the physiological conditions of different tissues and cells, and the distribution of adrenergic receptors in different cancer cells also has its own characteristics [28].

### Oncogene activation

The β-adrenergic signaling pathway stimulates several oncogenic signaling pathways, including those of Src and HER2 (encoded by ERBB2) [146]. In HER2-positive cells, the beta-adrenergic receptor activates signal transducer and activator of transcription 3 (STAT3), which in turn activates the ERBB2 promoter to stimulate gene transcription [147, 148]. The release of norepinephrine induced by beta-adrenergic receptors phosphorylates SCR S17 through the ADRB/cAMP/PKA axis, and further activates Y419 phosphorylation, leading to tumor growth [28]. Interestingly, in breast cancer, another study found that HER2 overexpression or continuous activation of ERK signaling can lead to epinephrine secretion in breast cancer cells, thereby upregulating the expression of the β2 receptor. The activation of β2 receptors can in turn promote the expression of HER2 mRNA and enhance its promoter activity [147]. This positive feedback pathway is crucial for the proliferation and migration of breast cancer cells.

An interesting study found that nervous stress induced the secretion of catecholamines, which are involved in the viral-related carcinogenic process [149]. In B-cell lymphoma and Kaposi’s sarcoma, β-adrenergic signaling induces PKA/cAMP response element binding protein (CREB) signaling and activates the Kaposi’s sarcoma-associated human herpesvirus 8 (HHV8) genome. The viral genome upregulates the expression of Rta, a major regulator of HHV8 [149]. This provides understanding of the molecular mechanism by which viruses can cause cancer.

## Targeting tumor innervation

Since the last century, researchers have attempted to cut nerves surgically to block tumor growth and metastasis (Table 1) [48, 50, 61, 94, 96–99, 102, 104, 150–155]. The use of surgical or chemical methods to cut off sympathetic adrenergic nerves can inhibit the occurrence of prostate cancer, and the blocking of parasympathetic cholinergic nerve signals can reduce the spread of prostate cancer cells [61]. Similarly, vagotomy or dermal sensory nerve ablation impairs the development of gastric or skin cancers (non-melanoma forms), respectively [48, 155]. Other methods, such as 6- hydroxydopamine (6HODA) and electrical stimulation, are also used to block neural input [28, 156]. However, these techniques have some shortcomings, that is, different tumor tissues have different innervation modes, such as stomach, pancreas, breast, and other secretory gland tumors, and the degree of innervation differs [122, 157, 158] thus, targeting neural input signals offers a promising approach in some specific tumors, but further exploration is needed in others.

**Table 1:** Beta-blockers mediate the tumor development in vivo.

Animal	Dose	Growth	Metastasis	Angiogenesis	Overall survival
BALB/c nude mice	1 μmol/100 g	Y	Y	/	/
BALB/c nude mice	100–800 mg	Y	Y	/	/
BALB/c nude mice	25 μM, 30 μl	Y	/	/	/
BALB/c nude mice	0.5 mg	/	Y	/	/
BALB/c nude mice	0.5 g/L	Y	Y	/	/
Athymic nude mice	2 mg/kg/d	Y	/	Y	/
BALB/c nude mice	10 mg/kg	Y	/	Y	/
SCID mice	10 mg/kg	Y	/	/	/
Organoid	25 uM	Y	/	/	Y
Organoid	25 uM	Y	/	/	Y
Kras + /LSL-G12D;Pdx1-Cre (KC) mice	/	Y	/	/	Y
BALB/c-Foxn1nu nude athymic mice	10 mg/kg/day	Y	/	/	/
Athymic BALB/c nude mice	5 mg/kg	Y	/	/	/
BALB/c nude mice	10 mg/kg	Y	/	Y	/
NOD/SCID mice	2 mg/kg/day	Y	/	/	/
SCID mice	2 mg/kg/day	Y	/	/	/
INS-GAS mice	/	Y	/	/	Y
INS-GAS mice	100U	Y	/	/	Y
Gli1; Ptch1 mice	/	Y	/	/	/

^*^Y: It has therapeutic effect; /: Not mentioned.

Many questions remain to be clarified regarding the regulation of the autonomic nervous system in tumor biology, especially in the parasympathetic nervous system. In the era of targeted therapies, radiation and chemotherapy combined with adjuvant therapeutic strategies, such as β-blocking, can provide a highly synergistic approach to control cancer progression (Table 2) [159–173]. In addition, the influence of psychosocial factors on tumors should not be underestimated. It has long been suspected that biological behaviors such as stress, depression, and social support can influence the development of cancer and disease progression, and the molecular mechanisms of these effects are now being explored [83]. Recent findings in laboratory models have suggested that biological behavior can directly influence the functional activity of cancer cells through the neuroendocrine system [97]. In a large-scale study investigating the relationship between psychological factors and tumorigenesis in 4825 subjects, chronic psychological stress was found to be associated with a high risk of tumorigenesis [174]. Stressful personalities, poor coping styles, negative emotional responses, and poorer quality of life are associated with higher cancer incidence, poor survival, and higher cancer mortality [85]. Chronic and acute stress are also predictors of recurrence in patients with cancer [175]. Interestingly, plasma epinephrine and norepinephrine levels fluctuate significantly during chronic and acute stress [176]. Similarly, in mouse models of prostate and ovarian cancers, stress and anxiety induced an increase in tissue catecholamines, which activated ADRB2/cAMP/PKA signaling and increased tumor growth and angiogenesis [94, 97]. However, it should be noted that, in addition to neuroendocrine hormones, peripheral nerves also play an important role. Researchers have found that some cancer tissues are highly innerved, and nerve cells in these tumors can release signaling factors in the form of exosomes to promote tumor growth [45].

**Table 2:** Clinical application of targeting innervation.

	Overall Survival	Incidence	Mortality	Metastasis	Recurrence
Prostate cancer	/	N	N and Y(ADT)	/	/
	/	/	Y	/	/
	/	/	N	/	/
	/	/	N	/	/
	N	/	N	/	/
Breast cancer	/	/	/	Y	Y
	/	N	Y	Y	/
	N	/	Y	/	/
	/	Y	Y	Y	Y
Lung cancer	Y	/	/	Y	/
	Y	/	/	/	/
Ovarian cancer	Y	/	/	/	/
	Y	/	/	/	/
Melanoma	/	/	/	/	Y
	Y	N	/	/	/
	Y	/	Y	/	Y

Note: Y, it has therapeutic effect; N, it has no therapeutic effect; /, Not mentioned.

Whether the use of antipsychotic drugs can prevent neural stress signals and thus inhibit tumor growth still needs to be further explored. Because fluoxetine is an antidepressant, it has anticancer effects in mice [177], but in some cases, it can also act directly on tumor cells to promote tumor cell proliferation [178]. It is worth mentioning that hyperglycemia, hyperlipidemia, advanced age, social support, stress, and depression can all alter the activity of the neuroendocrine system [83]. In addition to the direct activation of catecholamines, recent studies have reported that high-fat diet feeding and palmitic acid can increase the expression of β2AR via Sp1 in tumor tissues of CRC mice, and activation of cAMP/PKA increased the phosphorylation of hormone-sensitive lipase (HSL), a lipase that activates FFA, which is used as a fuel to provide energy for cancer growth, at S552 [179, 180]. Preclinical pharmacological studies are presently laying the groundwork for the translation of β-blockers as a novel adjuvant in clinical oncology to existing therapeutic strategies [181]. However, it is well-known that β-AR antagonists can exert severe side effects, including heart failure, bradycardia, prolonged hypoglycemia, bronchospasm, intermittent claudication, heart block, Raynaud’s phenomenon, and neurological reactions, including depression, fatigue, and nightmares [182, 183]. HSL inhibitors could be a viable alternative to βAR antagonists for CRC patients who experience psychological stress, are on a diet with high-fat content, or have other complications such as diabetes mellitus [180].

## Conclusions and remarks

Neural signaling has emerged as a therapeutic target after its role in cancer initiation and progression has been further understood [184, 185]. With advances in surgical techniques and a greater understanding of autonomic neuroanatomy, more precise denervation procedures have been developed.

Chemical denervation in pancreatic cancer has been effective at reducing clinically uncontrollable cancer pain, but as the authors state, this denervation is not permanent [186]. Repeated botulinum toxin injections are effective for the treatment of prostate cancer in mice [187]; however, this research did not translate into clinical success in humans (NCT01520441); thus, the frequency, dosage, and duration of denervation therapy need to be studied further. In the clinical treatment of gastric cancer, patients who underwent vagotomy combined with gastrectomy had a lower recurrence rate of gastric cancer than patients who underwent gastrectomy alone [155]. This suggests that denervation can be used as an adjuvant therapy to improve the success rate of surgical treatment.

In this review, we present evidence that activation of reprogramming signals and regenerative pathways to recruit nerve cells are important factors in the establishment and progression of cancers. The roles of the autonomic and sensory nerve groups in different tumor tissues may vary depending on the type of native tissue as well as the pattern of innervation of the native nerve. With recent advances in genetic engineering and imaging technologies, the role of nerve cells in the TME has progressed; however, many questions remain to be answered. For example; What factors are involved in neurogenesis and axonogenesis at different stages of tumorigenesis? How can we target tumor-specific pathways without affecting the established neural circuits elsewhere in the body? Will the nervous system mediate tumor initiation and development as feedback? Emerging technologies, such as electroceuticals, may help bridge this gap and provide minimally invasive tools [188–190]. Taken together, these data suggest that nerve aggregation is a novel feature of cancer, and that multiple surgical, pharmacological, and other approaches to interfere with nerve signaling in the TME represent a promising new strategy for cancer treatment.

## References

[1] Bray F, Ferlay J, Soerjomataram I, Siegel RL, Torre LA, Jemal A (2018). Global cancer statistics 2018: GLOBOCAN estimates of incidence and mortality worldwide for 36 cancers in 185 countries. CA Cancer J Clin.

[2] Erin N (2020). Role of sensory neurons, neuroimmune pathways, and transient receptor potential vanilloid 1 (TRPV1) channels in a murine model of breast cancer metastasis. Cancer Immunol Immunother.

[3] Welch DR (2006). Do we need to redefine a cancer metastasis and staging definitions?. Breast Dis.

[4] Zhao Z, Shilatifard A (2019). Epigenetic modifications of histones in cancer. Genome Biol.

[5] Monk M, Holding C (2001). Human embryonic genes re-expressed in cancer cells. Oncogene.

[6] Fraser SP, Diss JK, Chioni AM, Mycielska ME, Pan H, Yamaci RF (2005). Voltage-gated sodium channel expression and potentiation of human breast cancer metastasis. Clin Cancer Res.

[7] Bapat AA, Hostetter G, Von Hoff DD, Han H (2011). Perineural invasion and associated pain in pancreatic cancer. Nat Rev Cancer.

[8] Demir IE, Friess H, Ceyhan GO (2012). Nerve-cancer interactions in the stromal biology of pancreatic cancer. Front Physiol.

[9] Deshmukh SD, Willmann JK, Jeffrey RB (2010). Pathways of extrapancreatic perineural invasion by pancreatic adenocarcinoma: evaluation with 3D volume-rendered MDCT imaging. AJR Am J Roentgenol.

[10] Lei Y, Tang L, Xie Y, Xianyu Y, Zhang L, Wang P (2017). Gold nanoclusters-assisted delivery of NGF siRNA for effective treatment of pancreatic cancer. Nat Commun.

[11] Deng J, You Q, Gao Y, Yu Q, Zhao P, Zheng Y (2014). Prognostic value of perineural invasion in gastric cancer: a systematic review and meta-analysis. PLoS One.

[12] Espana-Ferrufino A, Lino-Silva LS, Salcedo-Hernandez RA (2018). Extramural perineural invasion in pT3 and pT4 gastric carcinomas. J Pathol Transl Med.

[13] Oven Ustaalioglu BB, Bilici A, Seker M, Kefeli U, Aydin D, Celik S (2019). Prognostic factors for operated gallbladder cancer. J Gastrointest Cancer.

[14] Cracchiolo JR, Xu B, Migliacci JC, Katabi N, Pfister DG, Lee NY (2018). Patterns of recurrence in oral tongue cancer with perineural invasion. Head Neck.

[15] Huyett P, Duvvuri U, Ferris RL, Johnson JT, Schaitkin BM, Kim S (2018). Perineural invasion in parotid gland malignancies. Otolaryngol Head Neck Surg.

[16] Schmitd LB, Scanlon CS, D’Silva NJ (2018). Perineural invasion in head and neck cancer. J Dent Res.

[17] Cui L, Shi Y, Zhang GN (2015). Perineural invasion as a prognostic factor for cervical cancer: a systematic review and meta-analysis. Arch Gynecol Obstet.

[18] Zhu Y, Zhang G, Yang Y, Cui L, Jia S, Shi Y (2018). Perineural invasion in early-stage cervical cancer and its relevance following surgery. Oncol Lett.

[19] Tan X, Sivakumar S, Bednarsch J, Wiltberger G, Kather JN, Niehues J (2021). Nerve fibers in the tumor microenvironment in neurotropic cancer-pancreatic cancer and cholangiocarcinoma. Oncogene.

[20] Wang H, Zheng Q, Lu Z, Wang L, Ding L, Xia L (2021). Role of the nervous system in cancers: a review. Cell Death Disco.

[21] Yin L, Li J, Wang J, Pu T, Wei J, Li Q (2021). MAOA promotes prostate cancer cell perineural invasion through SEMA3C/PlexinA2/NRP1-cMET signaling. Oncogene.

[22] Duraker N, Sisman S, Can G (2003). The significance of perineural invasion as a prognostic factor in patients with gastric carcinoma. Surg Today.

[23] Chen H, Liu D, Guo L, Cheng X, Guo N, Shi M (2018). Chronic psychological stress promotes lung metastatic colonization of circulating breast cancer cells by decorating a pre-metastatic niche through activating beta-adrenergic signaling. J Pathol.

[24] Huang Y, He L, Dong D, Yang C, Liang C, Chen X (2018). Individualized prediction of perineural invasion in colorectal cancer: development and validation of a radiomics prediction model. Chin J Cancer Res.

[25] Kinugasa T, Mizobe T, Shiraiwa S, Akagi Y, Shirouzu K (2017). Perineural invasion is a prognostic factor and treatment indicator in patients with rectal cancer undergoing curative surgery: 2000–11 data from a single-center study. Anticancer Res.

[26] Zare-Bandamiri M, Fararouei M, Zohourinia S, Daneshi N, Dianatinasab M (2017). Risk factors predicting colorectal cancer recurrence following initial treatment: a 5-year cohort study. Asian Pac J Cancer Prev.

[27] Ceyhan GO, Bergmann F, Kadihasanoglu M, Erkan M, Park W, Hinz U (2007). The neurotrophic factor artemin influences the extent of neural damage and growth in chronic pancreatitis. Gut.

[28] Zahalka AH, Frenette PS (2020). Nerves in cancer. Nat Rev Cancer.

[29] Hunt PJ, Kabotyanski KE, Calin GA, Xie T, Myers JN, Amit M. Interrupting neuron-tumor interactions to overcome treatment resistance. Cancers. 2020;12:3741. 10.3390/cancers12123741 PMC7762969 33322770

[30] Ding Y, Lee M, Gao Y, Bu P, Coarfa C, Miles B (2021). Neuropeptide Y nerve paracrine regulation of prostate cancer oncogenesis and therapy resistance. Prostate.

[31] Dubeykovskaya Z, Si Y, Chen X, Worthley DL, Renz BW, Urbanska AM (2016). Neural innervation stimulates splenic TFF2 to arrest myeloid cell expansion and cancer. Nat Commun.

[32] Yoneda T, Hiasa M, Okui T (2018). Crosstalk between sensory nerves and cancer in bone. Curr Osteoporos Rep.

[33] Pennington JW, Prentiss RJ, Howe G (1967). Radical prostatectomy for cancer: significance of perineural lymphatic invasion. J Urol.

[34] Hassan MO, Maksem J (1980). The prostatic perineural space and its relation to tumor spread: an ultrastructural study. Am J Surg Pathol.

[35] Rodin AE, Larson DL, Roberts DK (1967). Nature of the perineural space invaded by prostatic carcinoma. Cancer.

[36] Ayala GE, Wheeler TM, Shine HD, Schmelz M, Frolov A, Chakraborty S (2001). In vitro dorsal root ganglia and human prostate cell line interaction: redefining perineural invasion in prostate cancer. Prostate.

[37] Silverman DA, Martinez VK, Dougherty PM, Myers JN, Calin GA, Amit M (2021). Cancer-associated neurogenesis and nerve-cancer cross-talk. Cancer Res.

[38] Ayala GE, Dai H, Powell M, Li R, Ding Y, Wheeler TM (2008). Cancer-related axonogenesis and neurogenesis in prostate cancer. Clin Cancer Res.

[39] Zahalka AH, Arnal-Estape A, Maryanovich M, Nakahara F, Cruz CD, Finley LWS (2017). Adrenergic nerves activate an angio-metabolic switch in prostate cancer. Science.

[40] Wang L, Zeng H, Wang P, Soker S, Mukhopadhyay D (2003). Neuropilin-1-mediated vascular permeability factor/vascular endothelial growth factor-dependent endothelial cell migration. J Biol Chem.

[41] Kumanogoh A, Marukawa S, Suzuki K, Takegahara N, Watanabe C, Ch’ng E (2002). Class IV semaphorin Sema4A enhances T-cell activation and interacts with Tim-2. Nature.

[42] Kumanogoh A, Watanabe C, Lee I, Wang X, Shi W, Araki H (2000). Identification of CD72 as a lymphocyte receptor for the class IV semaphorin CD100: a novel mechanism for regulating B cell signaling. Immunity.

[43] Han H, Yang C, Zhang Y, Han C, Zhang G (2021). Vascular endothelial growth factor mediates the sprouted axonogenesis of breast cancer in rat. Am J Pathol.

[44] Zhao Q, Yang Y, Liang X, Du G, Liu L, Lu L (2014). The clinicopathological significance of neurogenesis in breast cancer. BMC Cancer.

[45] Amit M, Takahashi H, Dragomir MP, Lindemann A, Gleber-Netto FO, Pickering CR (2020). Loss of p53 drives neuron reprogramming in head and neck cancer. Nature.

[46] Saloman JL, Albers KM, Li D, Hartman DJ, Crawford HC, Muha EA (2016). Ablation of sensory neurons in a genetic model of pancreatic ductal adenocarcinoma slows initiation and progression of cancer. Proc. Natl Acad. Sci.

[47] Lucido CT, Wynja E, Madeo M, Williamson CS, Schwartz LE, Imblum BA (2019). Innervation of cervical carcinoma is mediated by cancer-derived exosomes. Gynecol Oncol.

[48] Peterson SC, Eberl M, Vagnozzi AN, Belkadi A, Veniaminova NA, Verhaegen ME (2015). Basal cell carcinoma preferentially arises from stem cells within hair follicle and mechanosensory niches. Cell Stem Cell.

[49] Chen H, Zhang W, Cheng X, Guo L, Xie S, Ma Y (2017). beta2-AR activation induces chemoresistance by modulating p53 acetylation through upregulating Sirt1 in cervical cancer cells. Cancer Sci.

[50] Eng JW, Reed CB, Kokolus KM, Pitoniak R, Utley A, Bucsek MJ (2015). Housing temperature-induced stress drives therapeutic resistance in murine tumour models through beta2-adrenergic receptor activation. Nat Commun.

[51] Mravec B, Horvathova L, Hunakova L. Neurobiology of cancer: the role of beta-adrenergic receptor signaling in various tumor environments. Int J Mol Sci 2020;21:7958. 10.3390/ijms21217958 PMC7662752 33114769

[52] Madeo M, Colbert PL, Vermeer DW, Lucido CT, Cain JT, Vichaya EG (2018). Cancer exosomes induce tumor innervation. Nat Commun.

[53] Vermeer PD, Bell M, Lee K, Vermeer DW, Wieking BG, Bilal E (2012). ErbB2, EphrinB1, Src kinase and PTPN13 signaling complex regulates MAP kinase signaling in human cancers. PLoS One.

[54] Adams RH, Wilkinson GA, Weiss C, Diella F, Gale NW, Deutsch U (1999). Roles of ephrinB ligands and EphB receptors in cardiovascular development: demarcation of arterial/venous domains, vascular morphogenesis, and sprouting angiogenesis. Genes Dev.

[55] Kojima T, Chang JH, Azar DT (2007). Proangiogenic role of ephrinB1/EphB1 in basic fibroblast growth factor-induced corneal angiogenesis. Am J Pathol.

[56] Wakhloo D, Scharkowski F, Curto Y, Javed Butt U, Bansal V, Steixner-Kumar AA (2020). Functional hypoxia drives neuroplasticity and neurogenesis via brain erythropoietin. Nat Commun.

[57] de Heer EC, Jalving M, Harris AL (2020). HIFs, angiogenesis, and metabolism: elusive enemies in breast cancer. J Clin Invest.

[58] Jing X, Yang F, Shao C, Wei K, Xie M, Shen H (2019). Role of hypoxia in cancer therapy by regulating the tumor microenvironment. Mol Cancer.

[59] Kobayashi T, Kihara K, Hyochi N, Masuda H, Sato K (2003). Spontaneous regeneration of the seriously injured sympathetic pathway projecting to the prostate over a long period in the dog. BJU Int.

[60] Dobrenis K, Gauthier LR, Barroca V, Magnon C (2015). Granulocyte colony-stimulating factor off-target effect on nerve outgrowth promotes prostate cancer development. Int J Cancer.

[61] Magnon C, Hall SJ, Lin J, Xue X, Gerber L, Freedland SJ (2013). Autonomic nerve development contributes to prostate cancer progression. Science.

[62] Metzdorf J, Hobloss Z, Schlevogt S, Ayzenberg I, Stahlke S, Pedreiturria X (2019). Fingolimod for irradiation-induced neurodegeneration. Front Neurosci.

[63] Lin Q, Shen F, Zhou Q, Huang P, Lin L, Chen M (2019). Interleukin-1beta disturbs the proliferation and differentiation of neural precursor cells in the hippocampus via activation of notch signaling in postnatal rats exposed to lipopolysaccharide. ACS Chem Neurosci.

[64] Lu Z, Elliott MR, Chen Y, Walsh JT, Klibanov AL, Ravichandran KS (2011). Phagocytic activity of neuronal progenitors regulates adult neurogenesis. Nat Cell Biol.

[65] Koizumi H, Higginbotham H, Poon T, Tanaka T, Brinkman BC, Gleeson JG (2006). Doublecortin maintains bipolar shape and nuclear translocation during migration in the adult forebrain. Nat Neurosci.

[66] Zhang J, Jiao J (2015). Molecular biomarkers for embryonic and adult neural stem cell and neurogenesis. Biomed Res Int.

[67] Kaplan MP, Chin SS, Fliegner KH, Liem RK (1990). Alpha-internexin, a novel neuronal intermediate filament protein, precedes the low molecular weight neurofilament protein (NF-L) in the developing rat brain. J Neurosci.

[68] Schult D, Holsken A, Buchfelder M, Schlaffer SM, Siegel S, Kreitschmann-Andermahr I (2015). Expression pattern of neuronal intermediate filament alpha-internexin in anterior pituitary gland and related tumors. Pituitary.

[69] Mauffrey P, Tchitchek N, Barroca V, Bemelmans AP, Firlej V, Allory Y (2019). Progenitors from the central nervous system drive neurogenesis in cancer. Nature.

[70] Engblom C, Pfirschke C, Zilionis R, Da Silva Martins J, Bos SA, Courties G, et al. Osteoblasts remotely supply lung tumors with cancer-promoting SiglecF(high) neutrophils. Science. 2017;358:eaal5081. 10.1126/science.aal5081 PMC6343476 29191879

[71] Lu R, Fan C, Shangguan W, Liu Y, Li Y, Shang Y (2017). Neurons generated from carcinoma stem cells support cancer progression. Signal Transduct Target Ther.

[72] Batlle R, Andres E, Gonzalez L, Llonch E, Igea A, Gutierrez-Prat N (2019). Regulation of tumor angiogenesis and mesenchymal-endothelial transition by p38alpha through TGF-beta and JNK signaling. Nat Commun.

[73] Ostrowski M, Carmo NB, Krumeich S, Fanget I, Raposo G, Savina A (2010). Rab27a and Rab27b control different steps of the exosome secretion pathway. Nat Cell Biol.

[74] Colombo M, Raposo G, Thery C (2014). Biogenesis, secretion, and intercellular interactions of exosomes and other extracellular vesicles. Annu Rev Cell Dev Biol.

[75] Kalluri R, LeBleu VS. The biology, function, and biomedical applications of exosomes. Science. 2020;367:eaau6977. 10.1126/science.aau6977 PMC7717626 32029601

[76] Liu H, Dong Y, Feng X, Li L, Jiao Y, Bai S (2019). miR-34a promotes bone regeneration in irradiated bone defects by enhancing osteoblastic differentiation of mesenchymal stromal cells in rats. Stem Cell Res Ther.

[77] Weng YS, Tseng HY, Chen YA, Shen PC, Al Haq AT, Chen LM (2019). MCT-1/miR-34a/IL-6/IL-6R signaling axis promotes EMT progression, cancer stemness and M2 macrophage polarization in triple-negative breast cancer. Mol Cancer.

[78] Yan X, Tang B, Chen B, Shan Y, Yang H, Reproducibility project: cancer b. replication study: the microRNA miR-34a inhibits prostate cancer stem cells and metastasis by directly repressing CD44. Elife. 2019;8:e43511. 10.7554/eLife.43511 PMC6414201 30860027

[79] Chua CEL, Tang BL (2019). miR-34a in Neurophysiology and Neuropathology. J. Mol. Neurosci.

[80] Salvo E, Campana WM, Scheff NN, Nguyen TH, Jeong SH, Wall I (2020). Peripheral nerve injury and sensitization underlie pain associated with oral cancer perineural invasion. Pain.

[81] Wirth LJ, Plotkin SR, Emerick KS, Cunnane ME, Faquin WC (2012). Case records of the Massachusetts General Hospital. Case 29-2012. A 49-year-old man with pain and cranial-nerve palsies after treatment of oral cancer. N Engl J Med.

[82] Bagan J, Sarrion G, Jimenez Y (2010). Oral cancer: clinical features. Oral Oncol.

[83] Antoni MH, Lutgendorf SK, Cole SW, Dhabhar FS, Sephton SE, McDonald PG (2006). The influence of bio-behavioural factors on tumour biology: pathways and mechanisms. Nat Rev Cancer.

[84] Armaiz-Pena GN, Cole SW, Lutgendorf SK, Sood AK (2013). Neuroendocrine influences on cancer progression. Brain Behav Immun.

[85] Chida Y, Hamer M, Wardle J, Steptoe A (2008). Do stress-related psychosocial factors contribute to cancer incidence and survival?. Nat Clin Pr Oncol.

[86] Glaser R, Kiecolt-Glaser JK (2005). Stress-induced immune dysfunction: implications for health. Nat Rev Immunol.

[87] Chrousos GP, Gold PW (1992). The concepts of stress and stress system disorders. Overv Phys Behav Homeost JAMA.

[88] Jiang L, Su H, Wu X, Shen H, Kim MH, Li Y (2020). Leptin receptor-expressing neuron Sh2b1 supports sympathetic nervous system and protects against obesity and metabolic disease. Nat Commun.

[89] Ye Y, Abu El Haija M, Morgan DA, Guo D, Song Y, Frank A (2020). Endocannabinoid receptor-1 and sympathetic nervous system mediate the beneficial metabolic effects of gastric bypass. Cell Rep.

[90] Wu WF, Wang L, Spetsieris N, Boukovala M, Efstathiou E, Brossner C, et al. Estrogen receptor beta and treatment with a phytoestrogen are associated with inhibition of nuclear translocation of EGFR in the prostate. Proc Natl Acad Sci. 2021;118:e2011269118. 10.1073/pnas.2011269118 PMC8020780 33771918

[91] Caparica R, Bruzzone M, Agostinetto E, De Angelis C, Fede A, Ceppi M (2021). Beta-blockers in early-stage breast cancer: a systematic review and meta-analysis. ESMO Open.

[92] Posielski NM, Richards KA, Liou JI, Borza T, Abel EJ, Downs TM, et al. Beta-adrenergic antagonists and cancer specific survival in patients with advanced prostate cancer: a veterans administration cohort study. Urology. 2021;155:186–191. 10.1016/j.urology.2021.02.008 33587939

[93] Oh MS, Guzner A, Wainwright DA, Mohindra NA, Chae YK, Behdad A (2021). The impact of beta blockers on survival outcomes in patients with non-small-cell lung cancer treated with immune checkpoint inhibitors. Clin Lung Cancer.

[94] Hassan S, Karpova Y, Baiz D, Yancey D, Pullikuth A, Flores A (2013). Behavioral stress accelerates prostate cancer development in mice. J Clin Invest.

[95] Madden KS, Szpunar MJ, Brown EB (2011). beta-Adrenergic receptors (beta-AR) regulate VEGF and IL-6 production by divergent pathways in high beta-AR-expressing breast cancer cell lines. Breast Cancer Res Treat.

[96] Sloan EK, Priceman SJ, Cox BF, Yu S, Pimentel MA, Tangkanangnukul V (2010). The sympathetic nervous system induces a metastatic switch in primary breast cancer. Cancer Res.

[97] Thaker PH, Han LY, Kamat AA, Arevalo JM, Takahashi R, Lu C (2006). Chronic stress promotes tumor growth and angiogenesis in a mouse model of ovarian carcinoma. Nat. Med.

[98] Pasquier E, Street J, Pouchy C, Carre M, Gifford AJ, Murray J (2013). beta-blockers increase response to chemotherapy via direct antitumour and anti-angiogenic mechanisms in neuroblastoma. Br J Cancer.

[99] Wolter JK, Wolter NE, Blanch A, Partridge T, Cheng L, Morgenstern DA (2014). Anti-tumor activity of the beta-adrenergic receptor antagonist propranolol in neuroblastoma. Oncotarget.

[100] Goldfarb Y, Sorski L, Benish M, Levi B, Melamed R, Ben-Eliyahu S (2011). Improving postoperative immune status and resistance to cancer metastasis: a combined perioperative approach of immunostimulation and prevention of excessive surgical stress responses. Ann Surg.

[101] Hasegawa H, Saiki I (2002). Psychosocial stress augments tumor development through beta-adrenergic activation in mice. Jpn J Cancer Res.

[102] Kim-Fuchs C, Le CP, Pimentel MA, Shackleford D, Ferrari D, Angst E (2014). Chronic stress accelerates pancreatic cancer growth and invasion: a critical role for beta-adrenergic signaling in the pancreatic microenvironment. Brain Behav Immun.

[103] Inbar S, Neeman E, Avraham R, Benish M, Rosenne E, Ben-Eliyahu S (2011). Do stress responses promote leukemia progression? An animal study suggesting a role for epinephrine and prostaglandin-E2 through reduced NK activity. PLoS One.

[104] Lamkin DM, Sloan EK, Patel AJ, Chiang BS, Pimentel MA, Ma JC (2012). Chronic stress enhances progression of acute lymphoblastic leukemia via beta-adrenergic signaling. Brain Behav Immun.

[105] Cole SW, Nagaraja AS, Lutgendorf SK, Green PA, Sood AK (2015). Sympathetic nervous system regulation of the tumour microenvironment. Nat Rev Cancer.

[106] Folkman J, Watson K, Ingber D, Hanahan D (1989). Induction of angiogenesis during the transition from hyperplasia to neoplasia. Nature.

[107] Eichmann A, Brunet I (2014). Arterial innervation in development and disease. Sci Transl Med.

[108] Carmeliet P, Tessier-Lavigne M (2005). Common mechanisms of nerve and blood vessel wiring. Nature.

[109] Nedvetsky PI, Zhao X, Mathivet T, Aspalter IM, Stanchi F, Metzger RJ (2016). cAMP-dependent protein kinase A (PKA) regulates angiogenesis by modulating tip cell behavior in a Notch-independent manner. Development.

[110] Zhao X, Nedvetsky P, Stanchi F, Vion AC, Popp O, Zuhlke K, et al. Endothelial PKA activity regulates angiogenesis by limiting autophagy through phosphorylation of ATG16L1. Elife. 2019;8:e46380. 10.7554/eLife.46380 PMC6797479 31580256

[111] De Bock K, Georgiadou M, Schoors S, Kuchnio A, Wong BW, Cantelmo AR (2013). Role of PFKFB3-driven glycolysis in vessel sprouting. Cell.

[112] Schoors S, De Bock K, Cantelmo AR, Georgiadou M, Ghesquiere B, Cauwenberghs S (2014). Partial and transient reduction of glycolysis by PFKFB3 blockade reduces pathological angiogenesis. Cell Metab.

[113] Petrova TV, Koh GY. Biological functions of lymphatic vessels. Science. 2020;369:eaax4063. 10.1126/science.aax4063 32646971

[114] Vaahtomeri K, Alitalo K (2020). Lymphatic vessels in tumor dissemination versus immunotherapy. Cancer Res.

[115] Bellingan GJ, Caldwell H, Howie SE, Dransfield I, Haslett C (1996). In vivo fate of the inflammatory macrophage during the resolution of inflammation: inflammatory macrophages do not die locally, but emigrate to the draining lymph nodes. J Immunol.

[116] Hu X, Deng Q, Ma L, Li Q, Chen Y, Liao Y (2020). Meningeal lymphatic vessels regulate brain tumor drainage and immunity. Cell Res.

[117] Farnsworth RH, Karnezis T, Maciburko SJ, Mueller SN, Stacker SA (2019). The interplay between lymphatic vessels and chemokines. Front Immunol.

[118] Bachmann SB, Gsponer D, Montoya-Zegarra JA, Schneider M, Scholkmann F, Tacconi C (2019). A distinct role of the autonomic nervous system in modulating the function of lymphatic vessels under physiological and tumor-draining conditions. Cell Rep.

[119] Allen JM, McHale NG, Rooney BM (1983). Effect of norepinephrine on contractility of isolated mesenteric lymphatics. Am J Physiol.

[120] McGeown JG, McHale NG, Thornbury KD (1987). The effect of electrical stimulation of the sympathetic chain on peripheral lymph flow in the anaesthetized sheep. J Physiol.

[121] McHale NG, Thornbury KD (1990). Sympathetic stimulation causes increased output of lymphocytes from the popliteal node in anaesthetized sheep. Exp Physiol.

[122] Le CP, Nowell CJ, Kim-Fuchs C, Botteri E, Hiller JG, Ismail H (2016). Chronic stress in mice remodels lymph vasculature to promote tumour cell dissemination. Nat Commun.

[123] Raju B, Haug SR, Ibrahim SO, Heyeraas KJ (2007). Sympathectomy decreases size and invasiveness of tongue cancer in rats. Neuroscience.

[124] Salmon H, Remark R, Gnjatic S, Merad M (2019). Host tissue determinants of tumour immunity. Nat Rev Cancer.

[125] Rudak PT, Choi J, Parkins KM, Summers KL, Jackson DN, Foster PJ (2021). Chronic stress physically spares but functionally impairs innate-like invariant T cells. Cell Rep.

[126] Cole SW, Arevalo JM, Takahashi R, Sloan EK, Lutgendorf SK, Sood AK (2010). Computational identification of gene-social environment interaction at the human IL6 locus. Proc Natl Acad Sci.

[127] Shahzad MMK, Arevalo JM, Armaiz-Pena GN, Lu C, Stone RL, Moreno-Smith M (2018). Stress effects on FosB and interleukin-8 (IL8)-driven ovarian cancer growth and metastasis. J Biol Chem.

[128] Bronte V, Kasic T, Gri G, Gallana K, Borsellino G, Marigo I (2005). Boosting antitumor responses of T lymphocytes infiltrating human prostate cancers. J Exp Med.

[129] Feig C, Jones JO, Kraman M, Wells RJ, Deonarine A, Chan DS (2013). Targeting CXCL12 from FAP-expressing carcinoma-associated fibroblasts synergizes with anti-PD-L1 immunotherapy in pancreatic cancer. Proc Natl Acad Sci.

[130] Qiao G, Bucsek MJ, Winder NM, Chen M, Giridharan T, Olejniczak SH (2019). beta-Adrenergic signaling blocks murine CD8(+) T-cell metabolic reprogramming during activation: a mechanism for immunosuppression by adrenergic stress. Cancer Immunol Immunother.

[131] Mohammadpour H, MacDonald CR, Qiao G, Chen M, Dong B, Hylander BL (2019). beta2 adrenergic receptor-mediated signaling regulates the immunosuppressive potential of myeloid-derived suppressor cells. J Clin Invest.

[132] Jena BC, Mandal M (2021). The emerging roles of exosomes in anti-cancer drug resistance and tumor progression: An insight towards tumor-microenvironment interaction. Biochim Biophys Acta Rev Cancer.

[133] Joyce JA, Fearon DT (2015). T cell exclusion, immune privilege, and the tumor microenvironment. Science.

[134] Niu Y, Lin Z, Wan A, Sun L, Yan S, Liang H, et al. Loss-of-function genetic screening identifies ALDOA as an essential driver for liver cancer cell growth under hypoxia. Hepatology. 2021;73:1461–1479. 10.1002/hep.31846 PMC8518375 33813748

[135] Burns-Cox N, Avery NC, Gingell JC, Bailey AJ (2001). Changes in collagen metabolism in prostate cancer: a host response that may alter progression. J Urol.

[136] Hisasue S, Kato R, Sato Y, Suetomi T, Tabata Y, Tsukamoto T (2005). Cavernous nerve reconstruction with a biodegradable conduit graft and collagen sponge in the rat. J Urol.

[137] Tuxhorn JA, Ayala GE, Smith MJ, Smith VC, Dang TD, Rowley DR (2002). Reactive stroma in human prostate cancer: induction of myofibroblast phenotype and extracellular matrix remodeling. Clin Cancer Res.

[138] Twardowski T, Fertala A, Orgel JP, San Antonio JD (2007). Type I collagen and collagen mimetics as angiogenesis promoting superpolymers. Curr Pharm Des.

[139] Egeblad M, Werb Z (2002). New functions for the matrix metalloproteinases in cancer progression. Nat Rev Cancer.

[140] Szpunar MJ, Burke KA, Dawes RP, Brown EB, Madden KS (2013). The antidepressant desipramine and alpha2-adrenergic receptor activation promote breast tumor progression in association with altered collagen structure. Cancer Prev Res.

[141] Hara MR, Kovacs JJ, Whalen EJ, Rajagopal S, Strachan RT, Grant W (2011). A stress response pathway regulates DNA damage through beta2-adrenoreceptors and beta-arrestin-1. Nature.

[142] Hara MR, Sachs BD, Caron MG, Lefkowitz RJ (2013). Pharmacological blockade of a beta(2)AR-beta-arrestin-1 signaling cascade prevents the accumulation of DNA damage in a behavioral stress model. Cell Cycle.

[143] Reeder A, Attar M, Nazario L, Bathula C, Zhang A, Hochbaum D (2015). Stress hormones reduce the efficacy of paclitaxel in triple negative breast cancer through induction of DNA damage. Br J Cancer.

[144] Kim J, Yu L, Chen W, Xu Y, Wu M, Todorova D (2019). Wild-type p53 promotes cancer metabolic switch by inducing PUMA-dependent suppression of oxidative phosphorylation. Cancer Cell.

[145] Montoya A, Varela-Ramirez A, Dickerson E, Pasquier E, Torabi A, Aguilera R (2019). The beta adrenergic receptor antagonist propranolol alters mitogenic and apoptotic signaling in late stage breast cancer. Biomed J.

[146] Armaiz-Pena GN, Allen JK, Cruz A, Stone RL, Nick AM, Lin YG (2013). Src activation by beta-adrenoreceptors is a key switch for tumour metastasis. Nat Commun.

[147] Shi M, Liu D, Duan H, Qian L, Wang L, Niu L (2011). The beta2-adrenergic receptor and Her2 comprise a positive feedback loop in human breast cancer cells. Breast Cancer Res Treat.

[148] Gu L, Lau SK, Loera S, Somlo G, Kane SE (2009). Protein kinase A activation confers resistance to trastuzumab in human breast cancer cell lines. Clin Cancer Res.

[149] Chang M, Brown HJ, Collado-Hidalgo A, Arevalo JM, Galic Z, Symensma TL (2005). beta-Adrenoreceptors reactivate Kaposi’s sarcoma-associated herpesvirus lytic replication via PKA-dependent control of viral RTA. J Virol.

[150] Campbell JP, Karolak MR, Ma Y, Perrien DS, Masood-Campbell SK, Penner NL (2012). Stimulation of host bone marrow stromal cells by sympathetic nerves promotes breast cancer bone metastasis in mice. PLoS Biol.

[151] Lin Q, Wang F, Yang R, Zheng X, Gao H, Zhang P (2013). Effect of chronic restraint stress on human colorectal carcinoma growth in mice. PLoS One.

[152] Palm D, Lang K, Niggemann B, Drell TLT, Masur K, Zaenker KS (2006). The norepinephrine-driven metastasis development of PC-3 human prostate cancer cells in BALB/c nude mice is inhibited by beta-blockers. Int J Cancer.

[153] Pasquier E, Ciccolini J, Carre M, Giacometti S, Fanciullino R, Pouchy C (2011). Propranolol potentiates the anti-angiogenic effects and anti-tumor efficacy of chemotherapy agents: implication in breast cancer treatment. Oncotarget.

[154] Renz BW, Takahashi R, Tanaka T, Macchini M, Hayakawa Y, Dantes Z (2018). beta2 adrenergic-neurotrophin feedforward loop promotes pancreatic cancer. Cancer Cell.

[155] Zhao CM, Hayakawa Y, Kodama Y, Muthupalani S, Westphalen CB, Andersen GT (2014). Denervation suppresses gastric tumorigenesis. Sci Transl Med.

[156] Thoenen H, Tranzer JP (1968). Chemical sympathectomy by selective destruction of adrenergic nerve endings with 6-Hydroxydopamine. Naunyn Schmiedebergs Arch Exp Pathol Pharmakol.

[157] Hayakawa Y, Sakitani K, Konishi M, Asfaha S, Niikura R, Tomita H (2017). Nerve growth factor promotes gastric tumorigenesis through aberrant cholinergic signaling. Cancer Cell.

[158] Schuller HM, Al-Wadei HA, Ullah MF, Plummer HK (2012). Regulation of pancreatic cancer by neuropsychological stress responses: a novel target for intervention. Carcinogenesis.

[159] Assayag J, Pollak MN, Azoulay L (2014). Post-diagnostic use of beta-blockers and the risk of death in patients with prostate cancer. Eur J Cancer.

[160] Aydiner A, Ciftci R, Karabulut S, Kilic L (2013). Does beta-blocker therapy improve the survival of patients with metastatic non-small cell lung cancer?. Asian Pac J Cancer Prev.

[161] Barron TI, Connolly RM, Sharp L, Bennett K, Visvanathan K (2011). Beta blockers and breast cancer mortality: a population- based study. J Clin Oncol.

[162] Botteri E, Munzone E, Rotmensz N, Cipolla C, De Giorgi V, Santillo B (2013). Therapeutic effect of beta-blockers in triple-negative breast cancer postmenopausal women. Breast Cancer Res Treat.

[163] Cardwell CR, Coleman HG, Murray LJ, O’Sullivan JM, Powe DG (2014). Beta-blocker usage and prostate cancer survival: a nested case-control study in the UK Clinical Practice Research Datalink cohort. Cancer Epidemiol.

[164] De Giorgi V, Gandini S, Grazzini M, Benemei S, Marchionni N, Geppetti P (2013). Effect of beta-blockers and other antihypertensive drugs on the risk of melanoma recurrence and death. Mayo Clin Proc.

[165] De Giorgi V, Grazzini M, Gandini S, Benemei S, Lotti T, Marchionni N (2011). Treatment with beta-blockers and reduced disease progression in patients with thick melanoma. Arch Intern Med.

[166] Diaz ES, Karlan BY, Li AJ (2012). Impact of beta blockers on epithelial ovarian cancer survival. Gynecol Oncol.

[167] Grytli HH, Fagerland MW, Fossa SD, Tasken KA (2014). Association between use of beta-blockers and prostate cancer-specific survival: a cohort study of 3561 prostate cancer patients with high-risk or metastatic disease. Eur Urol.

[168] Grytli HH, Fagerland MW, Fossa SD, Tasken KA, Haheim LL (2013). Use of beta-blockers is associated with prostate cancer-specific survival in prostate cancer patients on androgen deprivation therapy. Prostate.

[169] Lemeshow S, Sorensen HT, Phillips G, Yang EV, Antonsen S, Riis AH (2011). beta-Blockers and survival among Danish patients with malignant melanoma: a population-based cohort study. Cancer Epidemiol Biomark Prev.

[170] Posielski NM, Richards KA, Liou JI, Borza T, Abel EJ, Downs TM (2021). Beta-adrenergic antagonists and cancer specific survival in patients with advanced prostate cancer: a veterans administration cohort study. Urology.

[171] Powe DG, Voss MJ, Zanker KS, Habashy HO, Green AR, Ellis IO (2010). Beta-blocker drug therapy reduces secondary cancer formation in breast cancer and improves cancer specific survival. Oncotarget.

[172] Wang HM, Liao ZX, Komaki R, Welsh JW, O’Reilly MS, Chang JY (2013). Improved survival outcomes with the incidental use of beta-blockers among patients with non-small-cell lung cancer treated with definitive radiation therapy. Ann Oncol.

[173] Watkins JL, Thaker PH, Nick AM, Ramondetta LM, Kumar S, Urbauer DL (2015). Clinical impact of selective and nonselective beta-blockers on survival in patients with ovarian cancer. Cancer.

[174] Penninx BW, Guralnik JM, Pahor M, Ferrucci L, Cerhan JR, Wallace RB (1998). Chronically depressed mood and cancer risk in older persons. J Natl Cancer Inst.

[175] De Brabander B, Gerits P (1999). Chronic and acute stress as predictors of relapse in primary breast cancer patients. Patient Educ Couns.

[176] Schmidt C, Kraft K (1996). Beta-endorphin and catecholamine concentrations during chronic and acute stress in intensive care patients. Eur J Med Res.

[177] Hsu LC, Tu HF, Hsu FT, Yueh PF, Chiang IT (2020). Beneficial effect of fluoxetine on anti-tumor progression on hepatocellular carcinoma and non-small cell lung cancer bearing animal model. Biomed Pharmacother.

[178] Lei B, Xu L, Zhang X, Peng W, Tang Q, Feng C (2021). The proliferation effects of fluoxetine and amitriptyline on human breast cancer cells and the underlying molecular mechanisms. Environ Toxicol Pharm.

[179] Fatima S, Hu X, Gong RH, Huang C, Chen M, Wong HLX (2019). Palmitic acid is an intracellular signaling molecule involved in disease development. Cell Mol Life Sci.

[180] Fatima S, Hu X, Huang C, Zhang W, Cai J, Huang M (2019). High-fat diet feeding and palmitic acid increase CRC growth in beta2AR-dependent manner. Cell Death Dis.

[181] Cole SW, Sood AK (2012). Molecular pathways: beta-adrenergic signaling in cancer. Clin Cancer Res.

[182] Albouaini K, Andron M, Alahmar A, Egred M (2007). Beta-blockers use in patients with chronic obstructive pulmonary disease and concomitant cardiovascular conditions. Int J Chron Obstruct Pulmon Dis.

[183] Frishman WH (1988). Beta-adrenergic receptor blockers. Adverse effects and drug interactions. Hypertension.

[184] Chen D, Ayala GE (2018). Innervating prostate cancer. N Engl J Med.

[185] Amit M, Na’ara S, Gil Z (2016). Mechanisms of cancer dissemination along nerves. Nat Rev Cancer.

[186] Lillemoe KD, Cameron JL, Kaufman HS, Yeo CJ, Pitt HA, Sauter PK (1993). Chemical splanchnicectomy in patients with unresectable pancreatic cancer. A prospective randomized trial. Ann Surg.

[187] Coarfa C, Florentin D, Putluri N, Ding Y, Au J, He D (2018). Influence of the neural microenvironment on prostate cancer. Prostate.

[188] Spitzer NC (2015). Neurotransmitter switching? No surprise. Neuron.

[189] Yang B, Slonimsky JD, Birren SJ (2002). A rapid switch in sympathetic neurotransmitter release properties mediated by the p75 receptor. Nat Neurosci.

[190] Yamamori T, Fukada K, Aebersold R, Korsching S, Fann MJ, Patterson PH (1989). The cholinergic neuronal differentiation factor from heart cells is identical to leukemia inhibitory factor. Science.

